# Supplementary material for: Impact of Plastic-Related Compounds on P-Glycoprotein and Breast Cancer Resistance Protein In Vitro
Source: Molecules. 2023 Mar 17;28(6):2710. doi: 10.3390/molecules28062710 (PMC10058098; doi:10.3390/molecules28062710)
Supplement: Supplementary file 1 [file molecules-28-02710-s001.zip › molecules-2267277-supplementary.pdf]

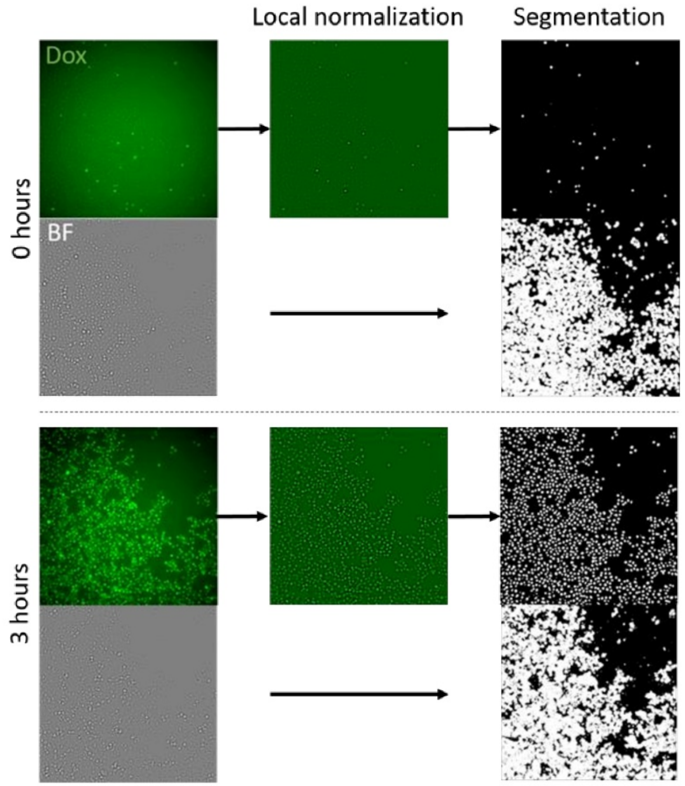
Local normalization
Segmentation
Dox
BF
0 hours
3 hours

Supplementary Figure S1. Image analysis workflow. Example frames from sample “CEM/ADR5000 cells + 20 μM doxorubicin + 4 × IC_{50} compound **5**” Fluorescence channel dox is first processed with local normalization to homogenize the field illumination and subsequently sent to Ilastik pixel classification workflow for segmentation of highly fluorescent cells (dox). Brightfield (BF) channel is directly segmented in Ilastik pixel classification workflow.

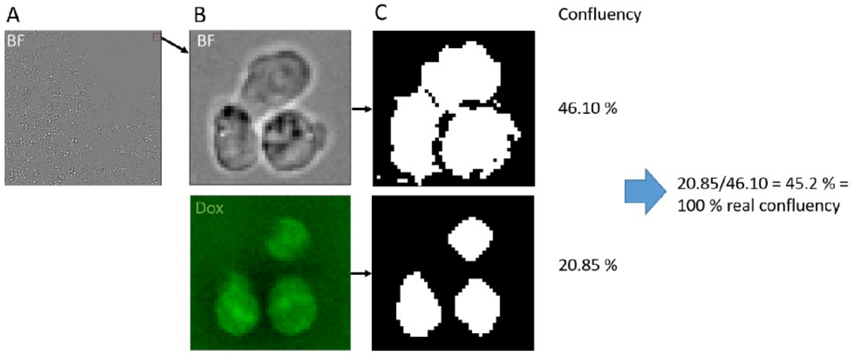

Supplementary Figure S2. Correction of fractional confluency analysis. (**A**) snapshot of brightfield channel of sample "CEM/ADR5000 cells + 20 µM doxorubicin + 4 × IC_{50} compound **5**". (**B**) Zoomed in region in brightfield and dox channels that contain 3 CEM/ADR5000 cells + 20 µM doxorubicin + 4 × IC_{50} compound **5**. (**C**) The corresponding segmentations and confluency correction.
