# Supplementary material for: Impact of Plastic-Related Compounds on P-Glycoprotein and Breast Cancer Resistance Protein In Vitro
Source: Molecules. 2023 Mar 17;28(6):2710. doi: 10.3390/molecules28062710 (PMC10058098; doi:10.3390/molecules28062710)
Supplement: Supplementary file 1 [file molecules-28-02710-s001.zip › Supplementary Video S1.pptx]

## Slide 1
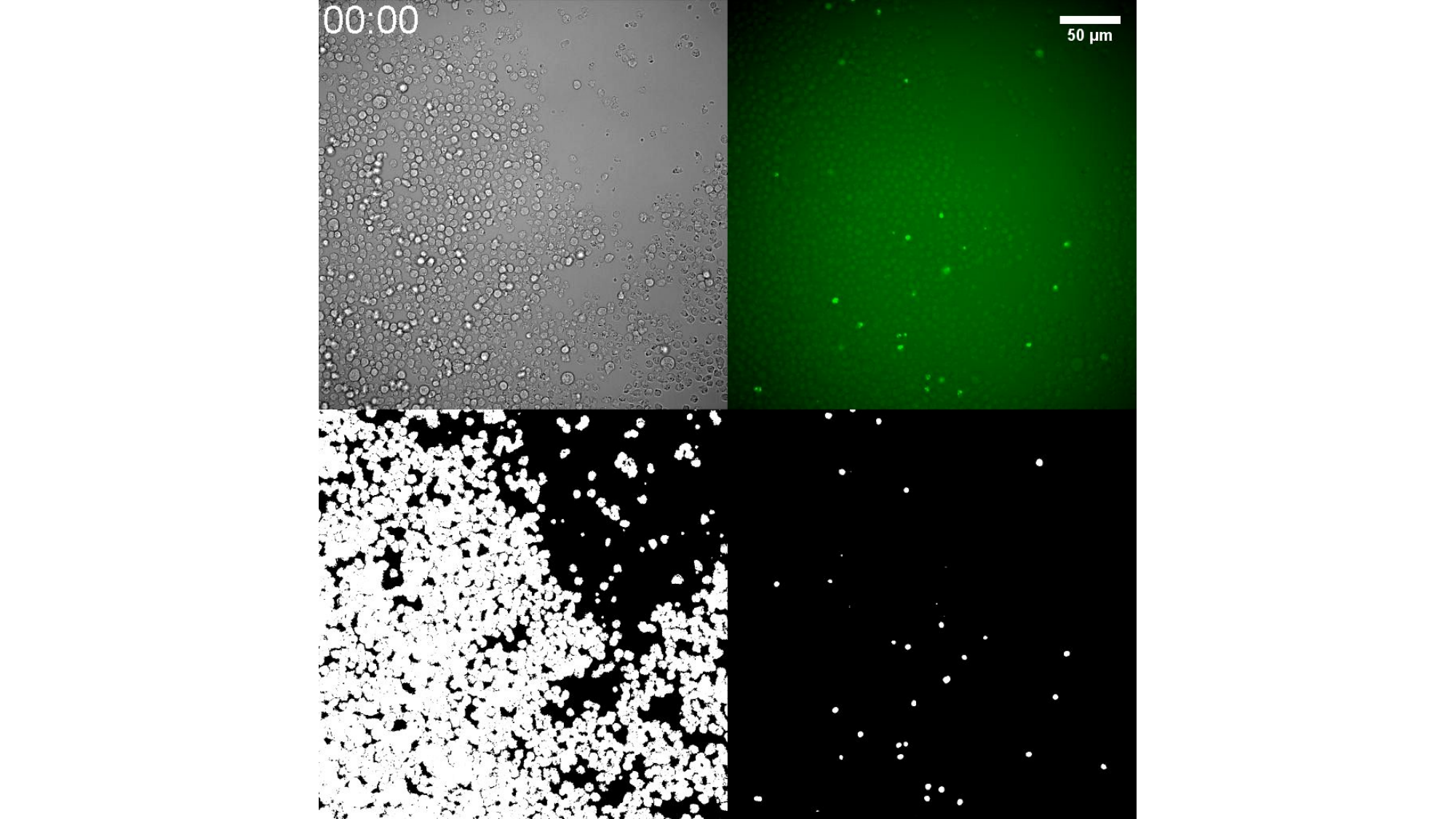

## Slide 2
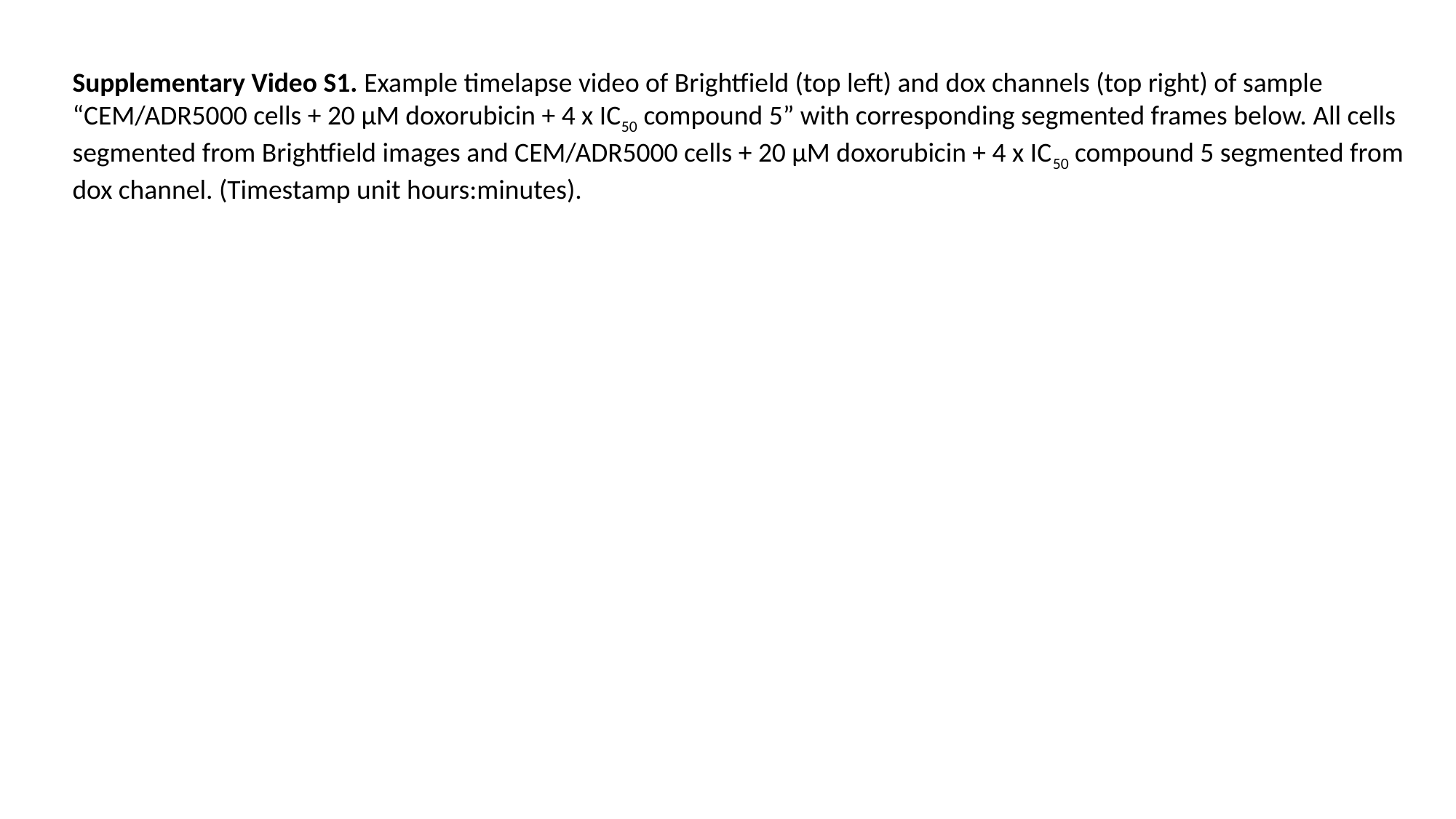

Supplementary Video S1. Example timelapse video of Brightfield (top left) and dox channels (top right) of sample “CEM/ADR5000 cells + 20 µM doxorubicin + 4 x IC50 compound 5” with corresponding segmented frames below. All cells segmented from Brightfield images and CEM/ADR5000 cells + 20 µM doxorubicin + 4 x IC50 compound 5 segmented from dox channel. (Timestamp unit hours:minutes).
